# From words to action: the development of the Neglected Tropical Disease Inclusion Score Card (NISC)

**DOI:** 10.1186/s40249-025-01340-6

**Published:** 2025-08-05

**Authors:** Robin van Wijk, Surya J. M. Baudoin, Bernice Ejiogu, Upasana Regmi, Mathias Duck, Ibrahim Rabiu, Constanze Vettel, Heleen Broekkamp, Roos Geutjes, Ruth M. H. Peters, Ida J. Korfage

**Affiliations:** 1 NLR | Until No Leprosy Remains, Amsterdam, the Netherlands; 2 Department of Public Health, Erasmus MC, University Medical Center Rotterdam, Rotterdam, the Netherlands; 3 Athena Institute, Vrije Universiteit Amsterdam, Amsterdam, the Netherlands; 4 Coventry University, Coventry, UK; 5 Lund University, Lund, Sweden; 6 The Leprosy Mission International, London, UK; 7 Department of Community Medicine, Gombe State University, Gombe, Nigeria; 8 DAHW German Leprosy and Tuberculosis Relief Association, Würzburg, Germany; 9 InfoNTD.Org, Amsterdam, the Netherlands

**Keywords:** Action Research, Inclusion, Neglected tropical diseases, Participation, Non-governmental organisations

## Abstract

**Background:**

The importance of patient participation in designing and delivering services for persons affected by neglected tropical diseases (NTDs) has gained increasing recognition. Responding to this, persons affected by NTDs urged NTD-focused non-governmental organisations (NGOs) to take action. These NGOs are pivotal in addressing healthcare disparities and reaching marginalised communities. To address the insufficient progress on participation, a participatory initiative was launched to develop a tool designed to support NGOs in fostering inclusion and ensuring the meaningful engagement of affected persons in their organisational decision-making processes.

**Methods:**

This research used an iterative, mixed-methods approach involving stakeholder input, semi-structured interviews, and surveys across two phases. Phase 1 included exploratory workshops with persons affected by NTDs and NTD NGO employees which led to the development of a first draft of a self-assessment tool. Phase 2 involved piloting the tool in NTD NGOs. Data were gathered via pre- and post-pilot interviews and surveys. Thematic analysis was used for the qualitative data and descriptive analysis for the quantitative data.

**Results:**

In phase 1, exploratory workshops revealed that meaningful participation involves creating environments where affected persons can openly share priorities and build their capacity. Workshop participants emphasised the need of inclusion at all stages of NGO activities. These insights informed the draft NTD Inclusion Scorecard (NISC), covering six domains. In phase 2, ten pilot sessions were conducted, feedback was gathered from 22 interviewees and 43 survey participants, focusing on the NISC’s usability and relevance. While feedback on the NISC was positive, participants highlighted the need for contextualisation, organisational commitment, and adding a communication domain to the NISC.

**Conclusions:**

The NISC is a self-assessment tool for NTD organisations, designed to enhance internal decision-making by fostering awareness of the importance of including the perspectives of persons affected by NTDs. By using the NISC, NGOs can identify gaps in inclusion and participation, improve their decision-making processes and provide services that are relevant and impactful for persons affected by NTDs. This tool provides insights that can guide NGOs in strengthening their role in promoting inclusion and increasing the effectiveness of their programmes.

**Graphical Abstract:**

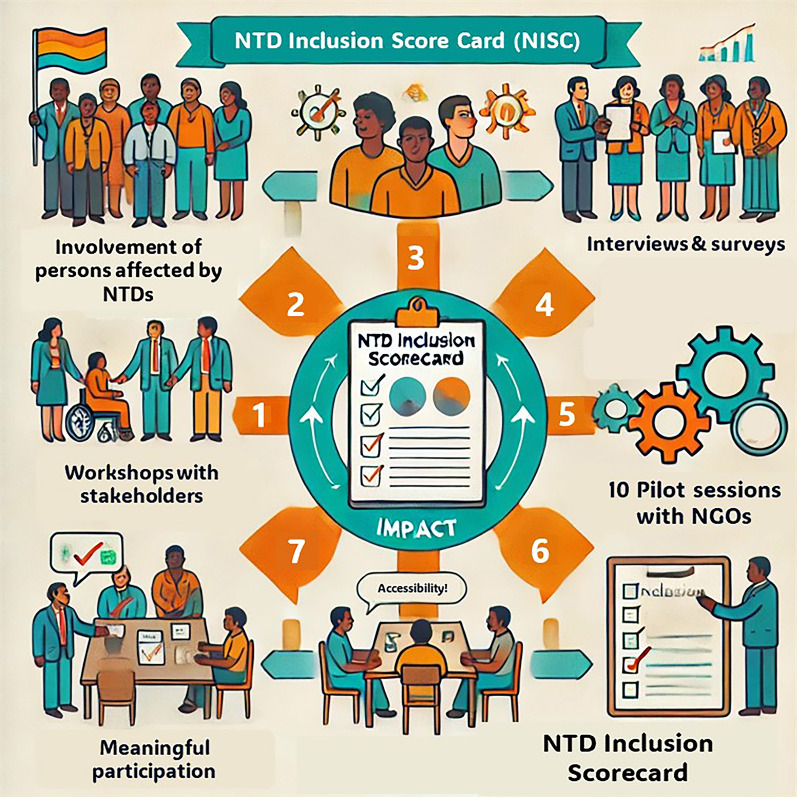

**Supplementary Information:**

The online version contains supplementary material available at 10.1186/s40249-025-01340-6.

## Background

Neglected tropical diseases (NTDs) are a group of twenty-one infectious diseases caused by various pathogens [1]. The World Health Organisation (WHO) estimates that globally over 1 billion people are affected by NTDs, and 1.6 billion people need prevention, treatment, or rehabilitation measures for these diseases [2–4]. NTDs cause irreversible impairments leading to disabilities, and have serious health, social, and economic impacts on the lives of those affected [5]. They place a significant strain on health systems with limited funds and negatively affect livelihoods, particularly in the most marginalised communities in tropical regions [6]. Many efforts to increase research and to address NTDs have been made by researchers, governments, non-governmental organisations (NGOs) and other organisations [2, 7]. Globally, the scope of initiatives to control and eliminate NTDs has expanded, focusing specifically on preventing, diagnosing, and treating these diseases [8]. Governments in regions endemic for NTDs may lack the resources or capacity to adequately address these diseases, leading to significant gaps in service delivery [9]. NGOs step in to fill these gaps by providing essential medical services, such as distributing medications, raising awareness, and training local healthcare workers [9, 10]. They reach remote communities that might otherwise be overlooked. However, most NGOs, working on NTDs, set up programmes and projects without consulting persons with NTDs, risking the chance of providing services that do not or not completely align with their needs [11].

According to persons affected by NTDs decision-making processes in governmental NTD programmes and NGOs seem to inadequately focus on the inclusion and participation of persons affected by NTDs [12]. It is important to put more attention on working with people experiencing NTD-related morbidity and disability, because these people are often underrepresented [2]. Swartz et al. and Souliotis et al. argue that involving persons from underrepresented groups can enhance decision-making outcomes by reducing biases, increasing creativity, and ensuring organisations hear their voices and consider their needs [13, 14]. Participation of persons affected with NTDs strengthens project ownership, effectiveness, and legitimacy and increases the range of people benefitting from projects [15, 16]. As shown by research, participation improves the quality and safety of healthcare services, enhances cost-effectiveness, and leads to better health outcomes [13, 17]. The WHO has developed guidelines on how patients’ voices should be heard in shared decision-making and health policy development about their own needs [18]. These guidelines emphasise assisting people in gaining control over the factors that affect their health and wellbeing. Consequently, patient participation has increasingly received attention in various aspects of healthcare processes and health policy development, aiming to improve patient safety, quality, and access to information [18, 19].

**Fig. 1:**
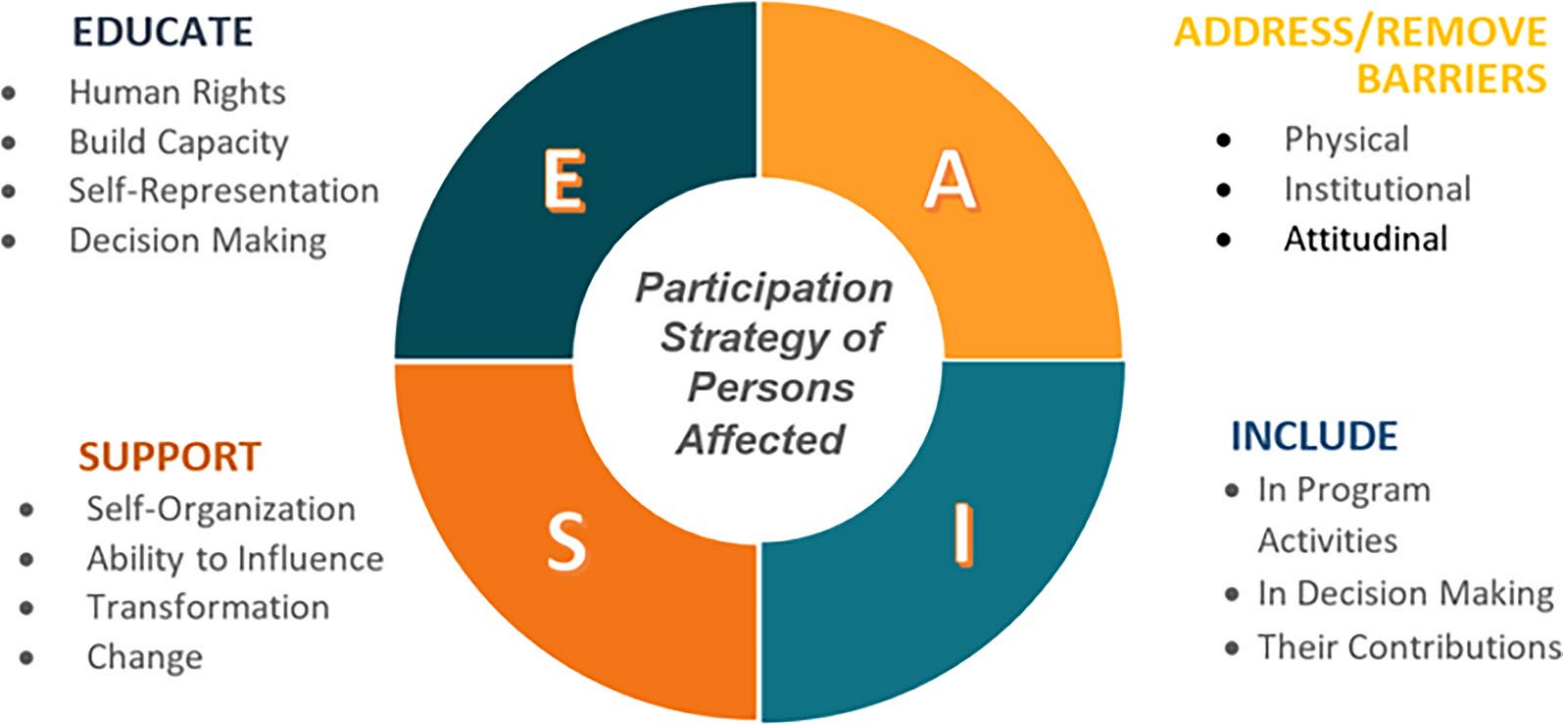
Educate, Adress barriers, support and include (EASI) Framework as a visualisation of the Statement of commitment to the participation of persons affected by neglected tropical diseases

Despite the increased attention for patient participation, patient participation in research, decision-making processes, and policy development this is challenging in practice [20]. For instance, Abma et al. studied patient involvement in nine research projects and discovered that the involvement of patients is mostly addressed only when mandated by the research funder [21]. Even then, participation often remained limited to a few activities, overlooking the set-up of activities or the design of follow-up phases [21]. To address the need for inclusion and participation and to tackle practical barriers, persons affected by NTDs united in 2019 during the annual NTD NGO Network (NNN) meeting to develop a statement on participation. This statement covers themes such as policy making, inclusive NTD programmes, leadership, networking, advocacy, and public representation. A visual representation can be found in Fig. 1, the full statement is available in the supporting information of this paper (Appendix 1). NGOs from the NNN and the International Federation of Leprosy Organisations (ILEP) endorsed this statement [22, 23]. However, despite this commitment, many organisations struggle with the practical implementation of actions. There was a recognised need from the NNN membership and ILEP to support NNN NGOs to uphold their commitment to the participation statement and increase accountability. In this context, a participatory trajectory was set up. The aim of this paper is to describe the development of a tool on how NTD NGOs can be supported to increase participation and inclusion of persons affected by NTDs in their organisations and decision-making processes.

## Methods

The study followed a participatory action research design, with a methodology of combining research and practical action to address the real-world challenge of participation of persons affected by NTDs in NTD NGOs [24]. This study followed a systematic collaborative process involving several key stakeholders, including NGO personnel, researchers and persons affected by NTDs. It was characterised by iterative action-reflection loops in the several phases of the tool development. The research group consisted of members of a sub-group of the Disease Management, Disability and Inclusion (DMDI) cross-cutting group connected to the NNN –two of the authors (RvW & MD) are active members of the DMDI cross-cutting group [12]. To ensure quality research, the current study adhered to the ‘Checklist for Assessing Quality in Action Research (QuARC)’ as proposed by Casey et al. [24] This checklist includes factors that should be considered when doing participatory action research and includes: context, quality of relationships, quality of the participatory action research process itself, and outcomes.

### Study design

**Fig. 2:**
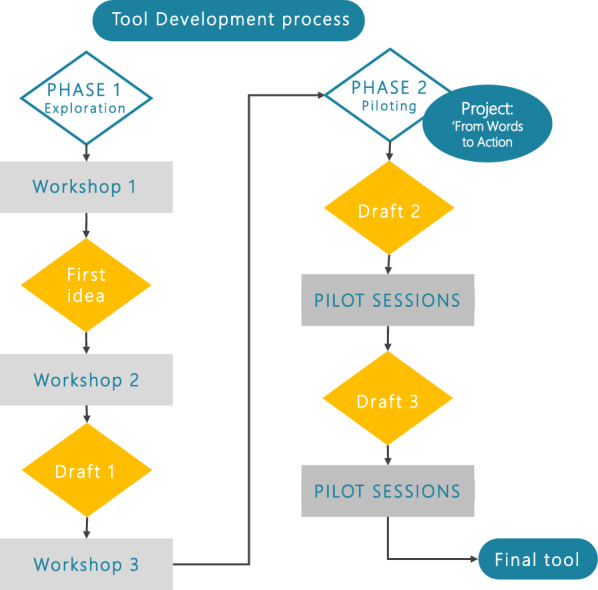
Study flow chart—Tool development process

An iterative, descriptive and mixed-methods approach was used in which stakeholder groups were heard through workshops, semi-structured interviews, and surveys. The study consisted of two consecutive phases which were initiated after the adoption of the statement of participation of persons affected by NTDs by the NNN community in 2019 (Fig. 2). The first phase consisted of three workshops involving NNN stakeholders and an inclusion expert. The second phase, also referred to as the “From Words to Action project”, focused on piloting several drafts of the designed tool. The latter allowed for an intensified development process, consisting of piloting and adjusting the tool according to a systematic process.

### Study population

For the first phase, the study population consisted of persons associated with NNN member organisations and persons affected by NTDs. These participants were recruited through convenience sampling within the network of the DMDI cross-cutting group. Particular attention was given to including organisations from various countries and focusing on different NTDs.

For the three workshops on generating ideas how to support NNN NGOs to uphold their commitment to the participation statement and increase accountability, there was no minimum or maximum number of participants. The inclusion criteria for participating in the workshops were as follows: (1) either being a person affected by an NTD or being a staff member of an NGO working in the NTD field; (2) being above the age of 18 years old; (3) having the ability to understand and speak English. There were no exclusion criteria.

For phase 2, the From Words to Action Project, the aim was to include at least ten NTD focused NGOs to pilot the draft tool that emerged from phase one. Pilot sessions were facilitated by a facilitator from outside the piloting organisation. This was often a member of the research team, although in some cases this was a hired expert from the country where the pilot was organised, to overcome language barriers. Throughout the pilot sessions, the aim was to include a selection of ten persons affected by an NTD and ten staff members for pre-and post-pilot interviews, resulting in an aimed sample size of 20 interview participants and 40 interviews. This would allow us to gain feedback on the tool from two different perspectives per pilot organisation. As the interviews did not include all pilot session participants, an additional survey was distributed to all participants involved in the pilot sessions, including both persons affected by NTDs and staff members. The inclusion criteria for the interview participants and survey respondents were as follows: (1) participation in the pilot session; (2) willing and able to give informed consent for participation in the interview; (3) either being a person affected by an NTD or being a staff member; (4) being above the age of 18 years old; for interviews only (5) having the ability to understand and speak English or Dutch (or a different language in presence of an independent translator); and 6) being available in both the pre-and post-interviews. There were no exclusion criteria.

### Data collection

#### Phase 1

Phase 1 was the initial phase of the project and involved exploratory workshops leading to the development of a first draft of the tool. Three workshops were organised during regular DMDI and NNN meetings and aimed to generate discussions about participation and inclusion of persons affected by NTDs among the participants. The workshops focused on how to keep organisations accountable, identifying necessary tools, and gathering feedback on the first draft tool. See Table 1 for a more detailed description of the workshops. Data were collected using digital boards with sticky notes and through note taking by the participating researchers. Because of the COVID-19 pandemic, all workshops took place online via Zoom.

**Table 1:** Detailed explanation of Phase 1 workshops

Workshop	Data collection
Workshop 1	Stakeholders were invited to an open brainstorm session focusing on identifying possible solutions to keep organisations accountable for their adherence to the ILEP/NNN statement on participation
Workshop 2	Stakeholders explored what the content of a self-assessment tool should entail
Workshop 3	Stakeholders were asked to provide feedback on an initial draft of the self-assessment tool, and the potential of the tool was further explored

#### Phase 2

In phase 2, pilot sessions were organised, and semi-structured interviews were conducted pre and post these pilot sessions. These were supplemented by surveys. The interview guides included questions about the interviewees’ perceptions of inclusion and participation to assess whether this perception aligned with how these concepts were included in the draft of the tool. Additional questions focused on the usability of the tool. Questions also focused on the progress of the pilot sessions and how to improve the tool. Pre-interviews were conducted before utilising the tool, while post-interviews and surveys were conducted after the use of the tool. These interviews and surveys were analysed to incorporate feedback into the third draft, leading to the final version of the tool. The interviews which mostly took place online via Zoom or Microsoft Teams, ranged from 30 to 60 min in duration. When needed, an independent translator was present to facilitate communication in the participant’s native language. The survey consisted of a minimum of 22 and a maximum of 44 items, depending on the participant’s domain affiliation. The questions included participants’ experiences during pilot sessions and feedback on the content of the tool. Most questions were multiple-choice that could be answered on a Likert scale. The survey was distributed via email and the software programme Qualtrics was used. It was available in English, French, and Indonesian. The second phase ran from November 2022 until January 2024.

### Data analysis

In phase 1, the digital boards with sticky notes and discussion notes were analysed through a combination of thematic analysis combined with discussions among the working group. First, the researchers (RvW, MD, HB) manually identified the most important themes discussed during the workshops through thematic analysis. Through this iterative process of alternating between workshops and analysis, decisions were made to lead to the draft of the tool used in the pilot sessions (start of phase 2 of this study).

During phase 2, all semi-structured interviews were video-recorded and automatically transcribed using Microsoft Teams or Zoom, transcripts were carefully checked and analysed by the research team. Subsequently, thematic deductive analysis with components of open coding was used to identify, analyse, and report patterns within the gathered data. The coding framework was based on an adjusted usability framework from the International Organisation for Standardisation (ISO) and existing codes from literature [25]. Coding was done with the software ATLAS.ti (by SJMB & UR) [26]. The survey responses were analysed using descriptive statistics with distributions (frequency) and measures of central tendency (mean) of the number of responses [27]. The answers to the open-ended questions were analysed in a similar way as the semi-structured interviews.

Interpretations and reflections have been discussed with the working group as well as with the later installed coordination committee overseeing the implementation of the pilot process.

### Ethics approval and consent to participate

The research ethics review committee of the Faculty of Science (BETHCIE) of the VU University in Amsterdam provided an ethics self-assessment check, of which the outcome was that explicit ethical approval was not considered necessary. Participants in Phase 1 were not asked to give informed consent, as no individual data were collected from them, and participation was based on their own interest. The participants in Phase 2 were all provided an information sheet about the project and were requested to sign an informed consent form. All data collected during the study were anonymised.

## Results

### Phase 1

#### Workshop 1: a self-assessment tool

The first workshop had twelve participants, including two persons affected by an NTD. The participants represented four continents: Africa, Asia, Europe, and South America. Participants represented NGOs focusing on Chagas disease, leprosy, lymphatic filariasis (LF) and trachoma. Three participants represented NGOs focusing on multiple NTDs.

Regarding the question ‘How can we achieve accountability by NNN member organisations?’, the group mentioned ‘*consider using a questionnaire, to ask NNN members about their current status*’. The group agreed that this questionnaire should be *‘simple and accessible for all members’*. Agreement was reached that a tool would be needed that ‘*can help NGOs keeping themselves accountable*’. The group concluded that ‘*a tool that can assess meaningful participation*’ would be needed, and it should ‘*provide guidelines on how to assess how persons affected by NTDs experienced their participation*’.

When discussing the question, ‘What are short-term and long-term changes we expect?’, the group agreed that experiences and examples of organisations that already focus on participation would be helpful to identify what practical steps can be taken by other organisations on a shorter term. It was also mentioned that not all persons affected by NTDs may have the capacity to provide input at the required professional level. However, NGOs would need to make efforts and provide opportunities for persons affected by NTDs to build their capacity and grow professionally on a longer term and to ‘*find a common level of understanding*’.

The workshop analysis identified themes including the suggestions for development of a questionnaire focused on NGO accountability. This led to the choice of a self-assessment tool to be used by NGOs. Based on existing knowledge and experiences from other NGOs working on inclusion, the Disability Inclusion Scorecard (DISC) was suggested as an example. This is a self-assessment tool that measures the extent of inclusion of persons with disabilities within all kinds of organisations [28, 29].

#### Workshop 2: towards an inclusion score card

The second workshop had seven participants, of which two were persons affected by an NTD. The participants represented three continents: Africa, Asia and Europe. A range of NTDs was represented through these participants, including leprosy, LF and trachoma. Two participants represented NGOs focusing on multiple NTDs.

**Fig. 3:**
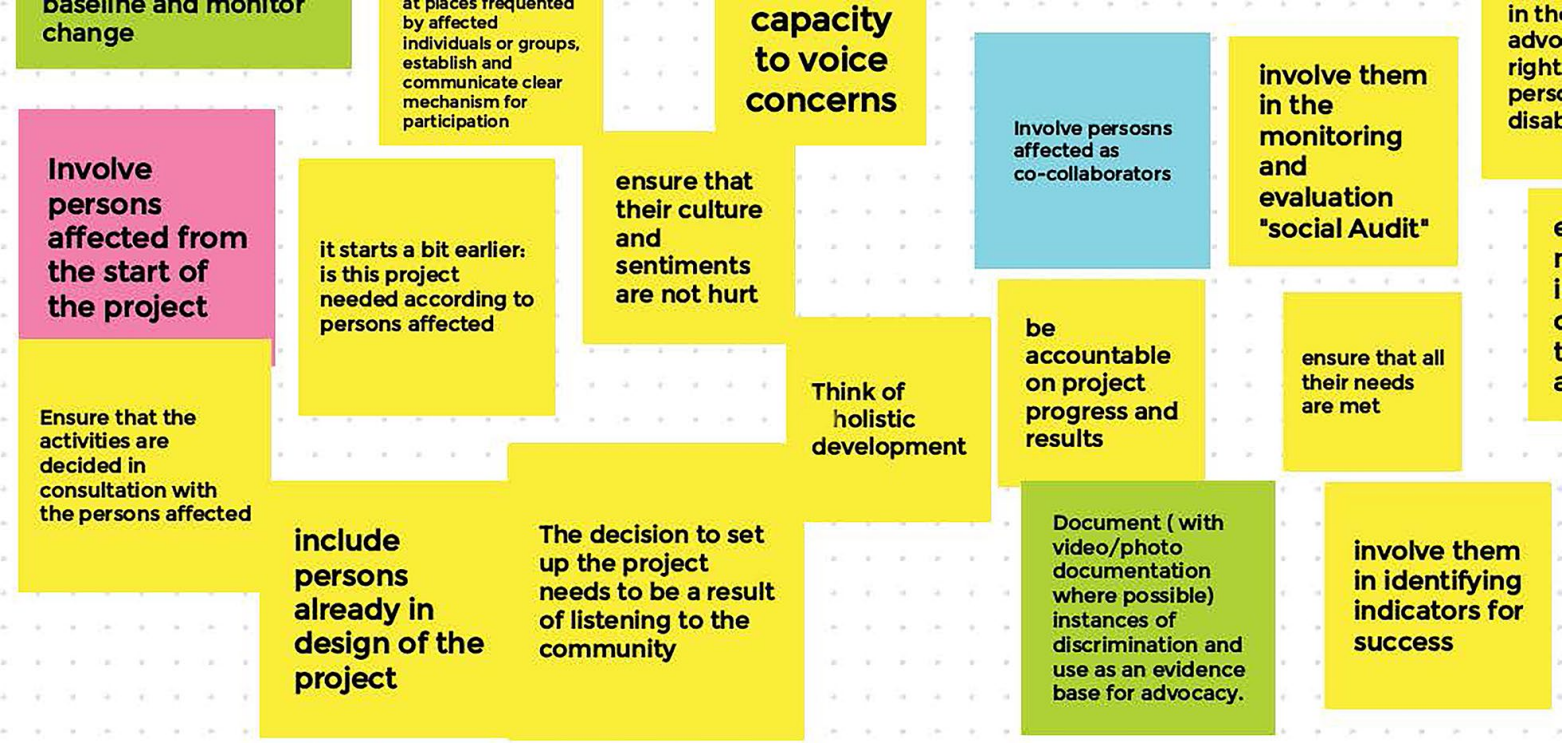
A sample of sticky note results reflecting on ‘what can an NGO do to ensure participation and inclusion of persons affected by NTDs’

Discussions on the question, ‘What is meaningful participation for persons affected by NTDs?’, also addressed how NGO leadership can facilitate such participation. It was mentioned that it is important ‘*to ensure an accessible space where persons affected can feel open to share their priorities*’. The group agreed that NGOs should create a safe environment and provide necessary support, including finances, transportation, and translation. They also agreed that difficult language, jargon and tokenism should be avoided. As mentioned in the first workshop, NGOs should also invest in building the capacity of persons affected by NTDs to participate in their organisational activities ‘*by ensuring more target beneficiaries are enrolled as employees*’. Workshop participants were clear about involving persons affected by NTDs in all stages of a project or process: ‘*involve persons affected from the start of the project, involve persons affected as collaborators*’ and ‘*be accountable for project progress and results*’. A small sample of the sticky note results reflecting on ‘what can an NGO do to ensure participation and inclusion of persons affected by NTDs’ can be found in Fig. 3.

The theme of accessibility was also discussed: ‘*Onboard them* [persons affected by an NTD] *first, so they know what it’s all about. Build their capacity and provide an interpreter so they can understand and speak*’. This is an example of how accessibility needs to be addressed by the organisation, including in terms of information and language accessibility. There was also attention to the fact that one or a few persons cannot represent a whole group: ‘*Create positions for people who work in a ‘bridge position’, who can engage larger communities of persons affected by NTDs*’.

The workshop participants responded positively to the set-up of the DISC and how the domains matched the themes discussed during the workshops. They appreciated how it ‘*focused on organisational change*’. However, the group acknowledged that the tool’s scope is too wide, focussing on all kinds of companies and organisations wanting to be more inclusive towards persons with a disability, and suggested that it should be adapted to focus more on the ‘*meaningful participation of persons affected and their experience*’ focussed on NTD organisations specifically. Other tools were also briefly discussed, but were not recommended by the workshop participants. It was agreed that the DISC would be the most suitable basis for developing a self-assessment tool regarding the aims and needs of the NNN community and persons affected by NTDs. The themes mentioned during the general discussions were linked to the different domains of the DISC. After this workshop, the research team in collaboration with persons affected by NTDs developed the first draft of the NTD Inclusion Score Card (NISC), consisting of questions in different domains: Governance, Programme Management, Human Resources, Financial Management, Accessibility, and External Relations.

#### Workshop 3: reflections on the first draft of the NTD inclusion score card

The third workshop was organised as part of the NNN conference in 2021. It attracted over 50 participants from multiple NNN member organisations, representing a wide range of NTDs. By dividing participants into five smaller groups, detailed feedback was gathered on the tool’s domains. Feedback ranged from the need for explanation of concepts to the relevance of questions to the NTD community.

##### General feedback

Participants shared their enthusiasm for the tool: ‘*Yes! The tool offers food for thought*’. And immediate discussion emerged about how the tool could be promoted and used by the wider NNN community: ‘*Use ‘% of NNN members who have done the NISC’ as an indicator*’. It was mentioned that NNN organisations vary greatly and the differences between the way international NGOs and more local NGOs work may not fit into one tool. ‘*The NISC possibly needs to be contextualised… this needs to be explained in a guide*’. Still, it was thought that it could be suitable for most NNN organisations and to ensure that the tool would be used and followed up in organisations, some commitment and discipline would be necessary. ‘*A champion within the organisation that takes up this issue and ensures embedding it in the structure of the organisation, following up on actions and scheduling assessments*’.

##### Feedback on the questions

The four scoring levels in the NISC questions were discussed by the group looking at the Governance domain. It was mentioned that sometimes participation could take the form of tokenism: ‘*Participation ladder, from tokenism to meaningful participation. These steps can be used for levels in these questions*’. Furthermore, it was recognised that expertise in human rights and inclusion may not be the same in every organisation. Which would also lead to differences in scoring. This continued into a discussion on the availability of internal or external knowledge on inclusion and participation and how this could possibly be reflected in the question’s scoring.

### Phase 2

For the interviews to assess relevance and usability of the first draft of the NISC, 22 interviewees were included, consisting of 14 male and eight female participants. Table 2 contains the characteristics of the interviewees. To gather additional input on how to improve the NISC, 43 survey respondents were recruited. Table 3 provides an overview of the characteristics of the survey respondents.

**Table 2:** Characteristics interview participants

Participant	Sex	Age, years	Education level	Representative	Focus NTD	Continent
P1	M	37	Tertiary	Project management	Onchocerciasis, trachoma	Africa
P2	M	30	Secondary	Person affected		Africa
P3	F	63	Tertiary	Project management	Leprosy	Asia
P4	F	26	Tertiary	Person affected and Project management	Leprosy	Asia
P5	M	38	Tertiary	Person affected	Leprosy	Asia
P6	F	39	Tertiary	Project management	LF, onchocerciasis, schistosomiasis, STH, trachoma	Africa
P7	M	48	Tertiary	Person affected and External relations	LF, onchocerciasis, schistosomiasis, STH, trachoma	Africa
P8	M	42	Tertiary	Person affected and Governance	LF, onchocerciasis, schistosomiasis, STH, trachoma	Africa
P9	M	48	Tertiary	Project management	LF, onchocerciasis, schistosomiasis, STH, trachoma	Africa
P10	M	64	Secondary	Person affected	LF, onchocerciasis, schistosomiasis, STH, trachoma	Africa
P11	F	55	Tertiary	Human resources	Leprosy	Europe
P12	F	Unknown	Tertiary	External relations	Leprosy	Europe
P13	F	61	Tertiary	Person affected	Leprosy	Europe
P14	M	30	Tertiary	Communication	Leprosy	Europe
P15	M	44	Tertiary	Person affected and External relations	Leprosy	Europe
P16	F	62	Tertiary	Governance	Leprosy	Africa
P17	M	35	Tertiary	Financial resources	LF, onchocerciasis, schistosomiasis, STH, trachoma	Africa
P18	M	43	Tertiary	Person affected	LF, onchocerciasis, schistosomiasis, STH, trachoma	Africa
P19	M	34	Tertiary	Financial resources	Leprosy and TB	Asia
P20	M	68	Tertiary	Person affected	Leprosy and TB	Asia
P21	F	Unknown		Governance	Leprosy	Asia
P22	M	Unknown		Person affected	Leprosy	Asia

*NTD* neglected tropical disease, *Person affected* a person affected by an NTD, *LF* lymphatic filariasis, *m* male, *f* female

**Table 3:** Characteristics survey respondents

Characteristics	Number of survey respondents (n)		
	Total	Draft 2	Draft 3
Persons affected by an NTD	**5**	**3**	**2**
Staff members	**38**	**24**	**14**
Governance	4	4	0
Programme management	22	14	8
Human resources	4	2	2
Financial resources	4	1	3
External relations	3	2	1
Communication	2	0	1
Continent			
Africa	24	15	9
Asia	6	4	2
Europe	13	8	5
Sex			
Female	18	10	8
Male	25	17	8

*NTD* neglected tropical disease

### Pilot sessions

During ten pilot sessions NGOs tested a draft version of the NISC. These each involved an average of seven participants, including employees and persons affected by an NTD. The average pilot session took approximately 3.5 hours, ranging from 1.5 to 6 hours. Although overall pilot participants experienced sufficient time to discuss every question (32/42), they preferred the session to be shorter. A diverse mix of participants from different organisational departments was perceived to positively impact the discussions: *‘The contribution of one* [colleague] *was more relevant than the other, but I think that we had the right composition … The different representation of the different domains was there.’* P11 [f, HR, Europe].

The facilitator was important for clarifying the exercise and questions: *‘The facilitation was very helpful because some questions were not easily understood and then the facilitator explained the meaning of the question, so I understood the question and could answer confidently.’* [m, Person affected, Asia]. Another important role of the facilitator was to ensure everyone participated equally. It was mentioned that equal participation was missing during one of the pilot sessions. Persons affected by NTDs, especially those who were not a staff member of the pilot organisations were observed to contribute less actively during pilot sessions than NTD-affected staff members. In contrast, an NTD-affected staff member was observed to freely express himself:

*‘During this discussion, we would rate ourselves. He* [interviewee talking about the person affected by an NTD in the pilot session] *had a way of saying, ‘No, I think it should be like this* [correcting the chosen level of inclusiveness of his team members]*. Because if you say it like that, it would mean that we are already doing this, meanwhile, you’re not doing this yet. It is to ensure that you don’t promote yourself beyond where you should be. So, it was beneficial, I would say.’* P6 [f, PM, Africa].

According to the interviewee, the person affected by an NTD dared to express his opinion, even when it was not in line with those of others. On the contrary, another interviewee, who participated in a session with a person affected by an NTD associated with the organisation, suggested that power dynamics might have influenced the varying contributions. An active contribution from a person affected by an NTD significantly relied on being able to talk in their language or having a translator present: *‘When you used his language then he* [person affected by an NTD] *was responding quite okay, quite positive, he was cooperating.’* P21 [f, G, Asia].

### General responses to the self-assessment tool

During the pre-pilot interviews, interviewees mentioned to expect that using the tool would contribute to evidence of inclusion and participation in the organisation and to guidelines on how to improve inclusion and participation. After seeing and using the tool, it was noted that opinions regarding the tool’s relevance differed across pilot organisations. Pilot participants from national NGOs were positive. However, the interviews revealed that international offices expressed doubts. Interviewees from international offices expressed their need to make the tool more generalisable and voiced reservations:

*‘Yes, I think it’s difficult, to what extent is it relevant to our organisation? In certain areas definitely, but for example, to hire someone with an NTD is very difficult because there are very few people who have [NTD] in our country.’—*P11 [f, HR, Europe].

The use of a self-assessment scorecard was positively perceived by most organisations. The majority saw the tool as a valuable initial step in assessing the inclusion and participation of persons affected by NTDs within their organisation. Interviewees and survey respondents would recommend it to other NTD organisations (31/39). Furthermore, most of the survey respondents (36/39) agreed that the tool was useful in identifying improvements in their organisation. Most respondents also found the tool to be visually appealing (29/42).

According to the survey respondents, persons affected by an NTD felt empowered to freely share their opinions (5/5). An interviewee expressed: ‘*Today I could speak freely; I can walk into the ministry of health and demand my right.*’ P18 [m, Person affected, Africa]. Another person affected by an NTD stated he could express his views on various aspects and have his voice heard: *‘I knew already about my rights as a person affected. But now seeing the questions from the tool, in reality, I feel that I’m still ignored in many things from the questions of the NISC.’* P5 [m, Person affected, Asia].

### Content of the tool

The majority of the survey respondents found the instructions clear (33/43) and the scoring method well explained (34/41). However, interviewees voiced uncertainties regarding the collaborative usage of the tool in a group setting. These uncertainties included whether discussions were needed, whether scoring should be individual or group-based, and whether responses should be specific to their own office or represent the entire organisation worldwide. It was also noted that some participants believed that the tool only served the purpose of usage at the implementation level and not the decision-making levels of the organisation.

**Fig. 4:**
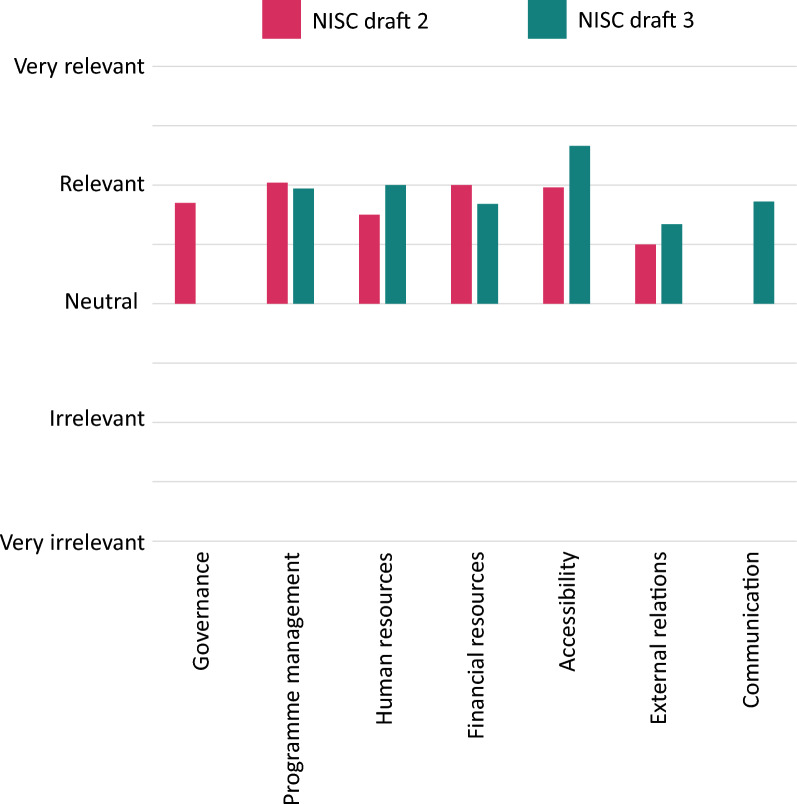
Relevancy questions per domain. Number of respondents per domain (draft 2: draft 3): Governance = 4:0, Project management = 14:8, Human resources = 2:2, Financial resources = 1:3, External relations = 2:1, Communication = 0:1

The different domains of the tool were considered to be relevant by most survey respondents (42/43; Fig. 4). Interviewees and survey participants both pointed out that only the domain of external relations lacked certain aspects, such as fundraising. Furthermore, both interview participants and survey respondents identified a need for the domain communication, therefore an additional domain focusing on communication was incorporated in the third draft of the NISC.

**Fig. 5:**
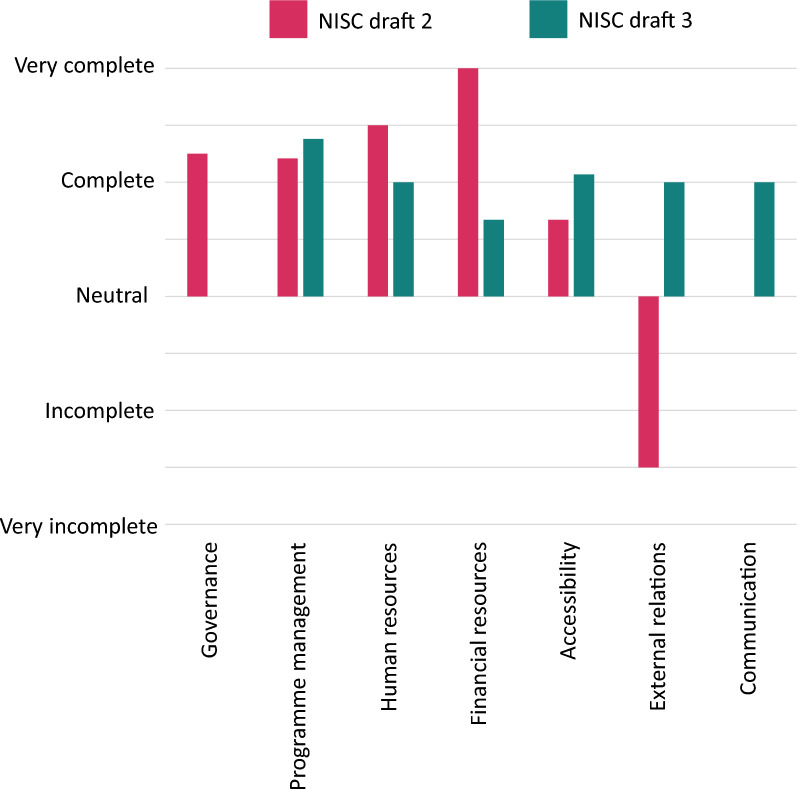
Completeness of the domains and questions. Number of respondents per domain (Draft 2: Draft 3): Governance = 4:0, Project management = 14:8, Human resources = 2:2, Financial resources = 1:3, External relations = 2:1, Communication = 0:1

Most respondents in both the interviews and the survey were positive about the content of the tool including the topics of the questions. Most respondents (36/43) found that they captured all aspects considering inclusion and participation in the respective domains. However, both interviewees and survey respondents expressed a preference for simpler wording to improve clarity. Slightly more than half of the survey respondents thought the tool was user-friendly (26/42). Figure 5 shows the survey outcomes regarding the experienced completeness of the domains. Moreover, the length of the tool and the length of the questions negatively affected user-friendliness, still some interviewees considered the detailed questions beneficial as explanations.

Regarding the answering options provided with the NISC questions, interviewees were less positive than survey respondents. They preferred simpler and shorter options. Multiple participants suggested using a colour code when choosing the options for the questions to enhance user-friendliness. None of the persons affected by NTDs expressed any concerns with the linguistic terms used in the tool. They felt included and satisfied during the session: *‘I felt like I was given attention from the NISC. The NISC wants to give attention to people affected in all aspects.’* P4 [f, Person affected-PM, Asia].

### Follow-up

Most organisations already made conscious efforts to foster the inclusion and participation of persons affected by an NTD in their organisation before participating in the pilots. Nonetheless, the majority did not have tangible action plans after the session. One of the interviewees was waiting for a follow-up when he was asked about the implementation of the action plan. Another stated that someone from senior management needed to take the lead in implementing the action plan after the session. According to one of the interviewees, the pilot session showed the value of having a senior manager participate and taking immediate action: *‘We had our country director as a participant and we all agreed on how we can make some changes in our work, it was good to have and I think it would be easier to implement the change or improvement.’* P9 [m, PM, Africa].

## Discussion

As part of how NTD NGOs can be supported in committing to the NNN/ILEP statement on the participation of persons affected by NTDs, this paper describes the process towards and the development of the NTD Inclusion Score Card (NISC). The tool aims to assess the extent of inclusion and participation of persons affected by NTDs in NTD NGOs. The NISC is not designed as a psychometric instrument or standardised measurement scale. It serves as a practical, reflective tool intended to support organisations working with persons affected by NTDs in initiating internal discussions on inclusion and in developing concrete action plans. It focuses on their programmes, but explicitly also on higher organisational levels, such as decision-making processes regarding governance, HR and finance. Besides, the NISC was mainly developed to push forward the conversation and foster a discussion by creating awareness within organisations and among their employees. This will support organisations in keeping themselves accountable for their commitment to the NNN/ILEP statement. The NISC is the first tool to assess the inclusion and participation of persons affected by NTDs within policy and decision-making processes in NGOs.

In the process towards the development of the NISC, a conscious decision was made to develop a self-assessment-through-group-discussion tool with a mix of employees from different departments and persons affected by NTDs. The choice for this approach was based on the assumption emerging during the workshops that an organisation would need to be intrinsically motivated to improve participation and inclusion of persons affected by NTDs and be willing to reflect critically on its efforts in achieving this. Dunning, Heath and Suls argue that self-assessment is often flawed, as people tend to overestimate themselves because they do not possess all the information necessary to reach an accurate self-assessment [30]. In addition, they state that those who perform poorly have the most difficulty ‘differentiating accurate from inaccurate performance’. While taking this into account, we argue that conducting the self-assessment through group discussions will help bring together the relevant knowledge by involving employees from different departments. These employees can challenge each other to think critically about the score they would give their organisation in the different domains. This is also related to the mix of participants from different organisational departments involved in the self-assessment exercise. This proved important for providing varied information. This is supported by the study of Van Nunen et al., who compared eight tools for assessing organisational cultures and showed that commitment at all levels of the organisation created ownership and cooperation from the participants [31]. Including persons affected by NTDs also provided different perspectives on the organisations level of inclusion.

To ensure meaningful participation of all people involved in the self-assessment exercise, one of the facilitating factors identified was to ensure a safe environment, where participants feel free to speak their mind. In the current study there was a noticeable variation in the level of contributions between different staff members, but also between NTD-affected staff members and those affected by NTDs from outside the organisation who were invited to participate in the NISC pilot sessions only. This effect was researched by Durand et al., who focused on the perceptions of group phenomena as determinants of self-assessed learning [32]. They state that psychological safety within the group is a strong predictor of organisational learning over time. They suggest that not only the learning outcomes but also reflexive discussions about the collaborative process are important to enhance an organisation’s performance. Based on this and the current study’s findings, it would therefore be essential to consider the power dynamics between participating members, which might contribute to people not freely speaking during the sessions.

The involvement of an external, neutral facilitator in the group discussions helps reduce self-overestimation and contributes to activating participants to meaningfully voice their opinions, which helps to maintain a balanced discussion. It is therefore recommended, in line with recommendations from the Center for Conflict Resolution, that the facilitator has excellent listening skills, challenges group thinking, raises questions to bring out different viewpoints and provides constructive feedback in case a person or people dominate the conversation [33]. In addition, the presence of an external, neutral facilitator will help to prevent misunderstandings about the goal of the session among the participants [34].

We found that NISC pilot participants expected that the NISC would include clear follow-up actions or guidelines linked to their assessment outcomes. However, during the pilot phase these were not yet available. A more general inclusion self-assessment tool that is used in all kinds of organisations, the Global Diversity and Inclusion Benchmarks (GDIB), offers guidance on effective strategies for improving diversity and inclusion in organisations. Additionally, the GDIB provides implementation guidelines to support organisations in translating these recommendations into actionable steps. They also include case studies and real-world examples of successful diversity and inclusion strategies already implemented by other organisations [35]. Ford and Evans also highlight that effective self-assessment tools include not only the measurement of an organisation, but also guides on where to look for solutions [36]. In response to the identified needs, additional features were added after the pilot phase ended. The NISC now also encourages an organisation to think about actions that can be taken to improve their score on each question. A specific space has been created to write out ideas for actionable steps, which can later be thought out more thoroughly as they can easily be shared through a Microsoft Word document. In addition, the research team has developed an ‘Action Plan Toolkit’ which includes guides and best practices sorted per NISC domain on how to follow-up on the assessment outcomes.

It is highly recommended that organisations develop an action plan within a short time frame after conducting the self-assessment. A lack of follow-up can be a barrier to effectively using the NISC. This potentially reduces the long-term sustainability of the tool, therefore also reducing the goal of addressing inclusion and participation. Samuelsson & Nilsson state that organisations that establish an action plan for the implementation after self-assessment carry it through more successfully than those that do not [34]. Furthermore, they show, just as suggested by participants for this study, that the commitment of top management is a success factor. For future research, it would also be recommended to follow-up and collect data on the NISC’s impact on organisational practice.

Overall, pilot participants were satisfied with the NISC, but the limited relevance of the tool perceived by NGOs with offices located in non-endemic countries stood out. These international NGOs usually have a bigger focus on fundraising in high income countries and mostly operate as a coordinator of international programmes in collaboration with national NGOs or branch offices located in endemic countries. For most of the employees of these organisations there are very few direct interactions with persons affected by NTDs, as these persons do not reside in their proximity. This may lead to these organisations thinking that efforts to include persons affected by NTDs in their decision-making processes are not relevant for them. However, when going through the NISC assessment process these international NGOs will find sufficient topics that apply to their specific situations in which they can include the views and opinions of persons affected by NTDs. This could for example be done through the establishment of a representative coalition of organisations of persons affected by NTDs [37]. A first step to build relationships with these kinds of coalitions can lead to consistent consulting and receipt of feedback on their organisational processes in non-endemic countries.

This study has several limitations that should be considered when interpreting the findings. Firstly, the overall number of participants involved in the project was relatively low, particularly in the quantitative component. While the qualitative element included a comparatively high number of participants, the limited size of the quantitative sample may affect the robustness of those specific findings. Secondly, the geographic scope of the study—while including participants from Asia, Europe, and Africa—was limited in its representation of other world regions, particularly Latin America and North America. Although an additional informal pilot was conducted with enthusiastic Latin American organisations after the formal study period, this was not captured as part of the research data through interviews or surveys and therefore is not included in the analysis. As a result, the global generalisability of the findings may be limited. Future research may assess the tool’s adaptability and perceived utility across diverse organisational and geographical settings. Larger-scale applications could provide opportunities for examining patterns of use and refining the tool based on user feedback and context-specific learning. Another limitation relates to the diversity of organisational staff members who responded to the survey. The majority of participants came from programme management departments, and recruiting staff from other departments proved more challenging than expected. This lack of diversity in professional background should be borne in mind, particularly when interpreting changes shown in Figs. 4 and 5 after the tool’s revision. A broader pool of respondents may have provided additional insights or resulted in different outcomes. Despite these limitations, the study provides important early insights into the relevance and applicability of the NISC tool in varied contexts, laying the groundwork for further adaptation and broader implementation.

## Conclusions

The NTD Inclusion Score Card (available via www.infontd.org/nisc) was developed through a participatory action research and has proven to be a promising tool to support NTD NGOs in improving the way persons affected by NTDs are included in their organisations and can participate in their decision-making processes. The NISC is a valuable addition to the existing initiatives focusing on patient participation, providing specific points of attention for NTD organisations regarding the inclusion and participation of persons affected by NTDs. In addition, the NISC pushes forward the conversation and fosters a discussion by creating awareness within organisations and among its employees. Commitment of employees from all departments and higher management is crucial in taking action based on the assessment outcomes. To maximise its impact, we recommend that all NGOs working with and for persons affected by NTDs explore the NISC’s usefulness in their context. Additionally, forming strong collaborations with organisations led by persons affected by NTDs should become standard practice among these organisations.

## Supplementary Information

Additional file 1.

## Data Availability

The datasets analysed during the current study are available from the corresponding author on reasonable request.

## Diseases and health conditions

NTD: Neglected tropical disease
LF: Lymphatic filariasis
Person affected: Person affected by an NTD

## Networks and organisations

NGO: Non-governmental organisation
NNN: NTD NGO network
ILEP: International federation of leprosy organisations
WHO: World Health Organisation
ISO: International organisation for standardisation
DMDI: Disease management, disability and inclusion
BETHCIE: Research ethics review committee of the faculty of science of the VU University

## Tools and Frameworks

NISC: NTD inclusion scorecard
DISC: Disability inclusion scorecard
GDIB: Global diversity and inclusion benchmarks
QuARC: Checklist for assessing quality in action research

## NISC Domains

G: Governance
PM: Project management
HR: Human resources
FR: Financial resources
ER: External relations
C: Communication
