# Supplementary material for: From words to action: the development of the Neglected Tropical Disease Inclusion Score Card (NISC)
Source: Infect Dis Poverty. 2025 Aug 5;14:80. doi: 10.1186/s40249-025-01340-6 (PMC12326779; doi:10.1186/s40249-025-01340-6)
Supplement: Supplementary file 1 — Additional file 1. [file 40249_2025_1340_MOESM1_ESM.pdf]

NEGLECTED TROPICAL DISEASE
NGO NETWORK
A global forum for nongovernmental organizations working together on NTDs

NNN Statement of commitment to the participation of persons affected by NTDs

Background

The involvement of communities and persons affected is not a new concept in primary health care and disease programmes. However, the active participation of persons affected by NTDs is not always a reality. Human rights-based approaches to neglected tropical diseases (NTDs) emphasise that any interventions should be based on the principles of participation, non-discrimination and accountability[1]. Participatory and human rights-based approaches, such as Disease Management, Disability and Inclusion (DMDI), address participation, non-discrimination and accountability as cross-cutting themes in NTD work. They are guided by the principle that engaging persons affected by NTDs is essential to the success of programmes. Having experienced the disease, disability and associated discrimination, persons affected by NTDs have a unique voice and perspective, they bring expertise and passion to the work and take the programmes closer to the communities they are designed to benefit. Aligning with these approaches helps to develop the Social Inclusion aspect of the NNN BEST Framework.

Call to action: the EASI strategy for participation of persons affected

To enable the active engagement of persons affected by NTDs, the NNN DMDI-Cross Cutting Group encourages all member organisations, partners and other stakeholders in the NTD community to adopt the EASI strategy to ensure that persons affected by NTDs have the space and the support for meaningful and effective participation.

[1] From "A human rights-based approach to neglected tropical diseases." https://www.who.int/gender-equity-rights/knowledge/ntd-information-sheet/en/ World Health Organization, 2010

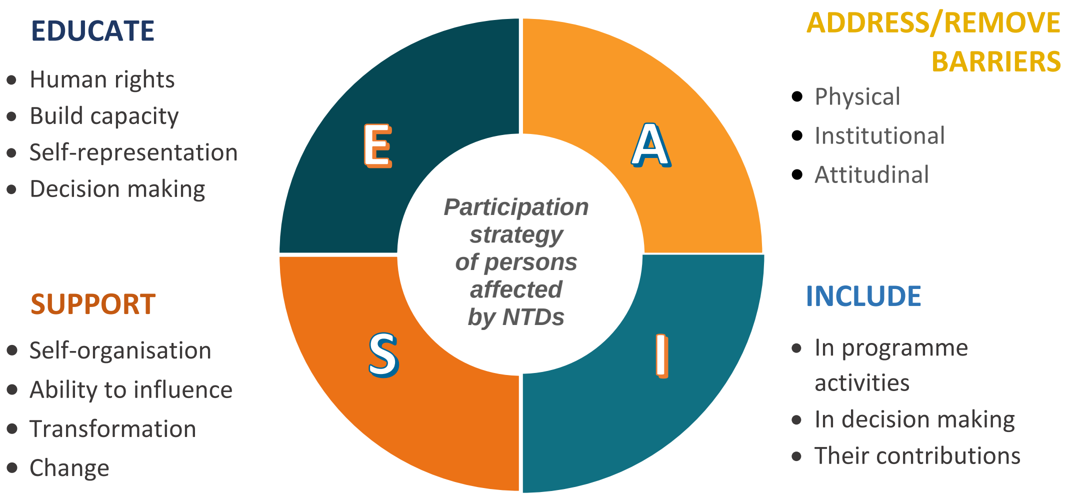

Our commitment

- **Policymaking**: Persons affected by NTDs are supported to participate in the development, monitoring and evaluation of NTD-related policies, guidelines and preferred practices at all levels.
- **Comprehensive and inclusive NTD programmes:** Persons affected by NTDs systematically participate in all stages of inclusive NTD programmes from planning, to implementation, monitoring and evaluation.
- **Leadership, networking and sharing:** Persons affected by NTDs are supported to take on leadership roles in self-care groups, support groups and other relevant local, national and international networks; to seek resources and build local capacity; to encourage participation of new members; and to share their experience with others.
- **Advocacy:** Persons affected by NTDs actively participate in NTD advocacy for the promotion of universal access to comprehensive and inclusive services that are fully integrated into strengthened local health systems.
- **Public representation:** Persons affected by NTDs are supported to actively participate at local, regional and global NTD fora and to become spokespersons in NTD campaigns or speakers at public events.
